# Concurrent Increases in Root Phosphorus Acquisition and Leaf Phosphorus Resorption Underpin Enhanced Phosphorus Economy in Chickpea During Reproductive Development

**DOI:** 10.1111/pce.70797

**Published:** 2026-08-09

**Authors:** Xiaolong Feng, Shuyan Li, Jiayin Pang, Peta L. Clode, Hans Lambers, Kadambot H. M. Siddique

**Affiliations:** ^1^ School of Biological Sciences The University of Western Australia Perth Western Australia Australia; ^2^ The UWA Institute of Agriculture The University of Western Australia Perth Western Australia Australia; ^3^ Centre for Microscopy, Characterisation and Analysis The University of Western Australia Perth Western Australia Australia

**Keywords:** leaf acid phosphatase activity, leaf P fraction, nitrogen fixation, phosphorus‐resorption efficiency, photosynthetic P‐use efficiency, reproductive demand

## Abstract

Phosphorus (P) acquisition and leaf P‐use strategies are critical for reproductive success and productivity, yet, their developmental association during the reproductive phase remains unclear. Five chickpea accessions contrasting in P‐remobilisation efficiency (PRE) were grown in a temperature‐controlled glasshouse under moderate P deficiency. Root P‐acquisition traits (rhizosheath carboxylates and rhizosheath acid phosphatase activity) and leaf P‐use traits (PRE, photosynthetic P‐use efficiency (PPUE), P fractions, and leaf acid phosphatase activity) were measured. Phosphorus acquisition substantially increased during reproduction compared with the vegetative stage, with greater carboxylates recovered from the rhizosheath and higher rhizosheath acid phosphatase activity. Root nitrogen (N) and P concentrations increased by 108% and 54%, respectively. Leaf PRE increased by 28%, with higher resorption efficiency across most P fractions, whereas N‐resorption efficiency was stable. Although area‐based photosynthetic rate increased by 34%, PPUE did not, and we observed no trade‐off between PPUE and PRE. Leaf thickness and leaf mass per area increased by 22% and 34%, respectively, partially explaining the faster photosynthetic rate. Concurrent we also observed increases in transpiration, and this may be associated with increased photosynthetic capacity. These results reveal coordinated increases in root P acquisition and leaf P remobilisation during reproduction, likely driven by stronger sink demand.

## Summary statement

- At its reproductive stage, chickpea simultaneously enhances both root phosphorus (P) acquisition and leaf internal P remobilisation, demonstrating coordinated P‐use strategies.
- Leaf photosynthetic P‐use efficiency remains stable while P‐resorption efficiency increases, indicating no trade‐off between P‐use and P‐conservation strategies.

## 1 Introduction

With the ongoing depletion of phosphate rock, enhancing phosphorus (P)‐use efficiency (PUE) and reducing dependence on P fertilisers have become increasingly important, especially for crop production (Feng et al. 2026; Veneklaas et al. 2012). Chickpea – the third most widely cultivated legume globally and a major cool‐season crop grown on marginal soils in semi‐arid regions – plays a crucial role in providing high‐quality protein for human diets, particularly in developing regions (FAO 2020). It is widely cultivated in the Mediterranean, Australia, India and Ethiopia, and its importance is expected to increase under climate change (Varshney et al. 2021). However, P deficiency remains a major constraint for chickpea growth and yield (Srinivasarao et al. 2006).

Improving PUE can be achieved either by increasing the acquisition of external P sources and/or by enhancing the efficiency of internal P utilisation. In recent decades, significant efforts have focused on improving plant P acquisition from soil, including the development of crops with modifying root architecture, increasing secretion of root exudates that solubilise soil‐bound P, and enhancing symbiotic interactions with mycorrhizal fungi (Dissanayaka et al. 2018; Lambers 2022; Lambers et al. 2015; Veneklaas et al. 2012). Furthermore, breeding programs and biotechnological innovations have focused on engineering P transporters and regulatory networks to improve P‐acquisition efficiency, as highlighted in recent comprehensive reviews (Guo et al. 2025; Paz‐Ares et al. 2022; Wen et al. 2022; Yang et al. 2024).

Sustainable and productive cropping systems require a balance between P inputs and outputs; thus, increasing root P acquisition while improving internal P redistribution and utilisation are essential for achieving high yields per unit P uptake (Feng et al. 2026; Veneklaas et al. 2012). However, the coordination between aboveground P‐use strategies (e.g., P resorption and PPUE) and belowground P acquisition remains poorly understood. Therefore, more attention should be directed toward enhancing P retranslocation and reutilisation within plants (e.g., P resorption), rather than focusing solely on root P acquisition (Dissanayaka et al. 2018; Nadeem et al. 2022; Smith et al. 2015).

Phosphorus resorption plays a crucial role in internal P cycling within plants. By recycling P from senescing organs, plants reduce their reliance on external P sources, thereby enhancing PUE. Phosphorus resorption can meet 24%–48% of plant P demand, with higher contributions in ecosystems with low soil P availability (Cleveland et al. 2013; Sardans et al. 2017). In a mature *Eucalyptus* woodland, annual P resorption accounts for 45% of total plant nutrient demand and approximately 80% of annual P uptake at the ecosystem level (Jiang et al. 2024). At the individual plant level, up to 60% of P required for growth may be recovered through resorption from senescing organs (Veneklaas 2022). Under low‐P conditions, up to 81% of wheat grain P is derived from remobilisation from senescing vegetative organs, and interrupting P supply post‐anthesis promotes P remobilisation from leaves and other vegetative organs without significantly affecting grain yield (El Mazlouzi et al. 2020; El Mazlouzi et al. 2020). Higher P‐resorption efficiency (PRE) confers a competitive advantage in P‐limited environments, as redistributed P supports new organ formation, reproductive success and overall plant growth (Veneklaas et al. 2012; Tully et al. 2013). However, despite its importance, the regulation of P resorption and its coordination with root P acquisition during reproductive development remain poorly understood, limiting our understanding of how plants integrate internal P remobilisation with external P uptake and, consequently, our ability to enhance whole‐plant P‐use economy.

Photosynthetic P‐use efficiency (PPUE) is a critical component of overall PUE. Proteaceae from severely P‐improverished habitats preferentially allocate P to mesophyll cells, thereby increasing their PPUE (Hayes et al. 2018). In contrast, chickpea improves PPUE by reducing leaf inorganic phosphate (P_i_) and metabolite P concentrations, while increasing the proportion of P allocated to nucleic acid P, rather than preferentially allocating P to mesophyll cells (Wen et al. 2023). Such adjustments of leaf P fractions may influence PRE, as organic P must be hydrolysed into P_i_ before remobilisation (Aerts 1996; Fischer 2007; Hidaka and Kitayama 2011). Inorganic P is highly mobile in plants and can be readily resorbed during senescence (Dhakal et al. 2025). Therefore, increasing PPUE by reducing P_i_ allocation in leaves may constrain P resorption, leading us to hypothesise a trade‐off between PPUE and PRE in chickpea.

Nutrient demand at different developmental stages can influence resorption efficiency. During periods of rapid growth or reproductive development, increased internal nutrient demand may enhance nutrient resorption (Avila‐Ospina et al. 2014; Gerdol et al. 2019; Karlsson 1994; Tully et al. 2013). Species with greater reproductive investment often exhibit increased nutrient resorption and efficient internal redistribution. For example, female Euphrates poplar (*Populus euphratica*) individuals exhibit higher PRE than males (Yu et al. 2022). Similarly, Pracaxi trees (*Pentaclethra macroloba*) with greater reproductive demands show higher leaf N and P resorption proficiency than those with lower reproductive demands, with the highest N‐resorption efficiency (NRE) and PRE observed in stands growing on moderately fertile soils with higher reproductive demands rather than those on P‐impoverished soils (Tully et al. 2013). However, some studies report no clear evidence that reproductive investment is compensated through nutrient resorption from senescing leaves (Eckstein and Karlsson 2001; Niva et al. 2003), suggesting that these species may rely more on soil nutrient uptake. Previous studies have shown that rhizosheath carboxylate concentration – a key root P‐acquisition strategy – increases significantly during the reproductive stage compared with the vegetative stage in chickpea (Pang et al. 2022; Wouterlood et al. 2004). Whether this developmental increase in belowground P acquisition is accompanied by changes in aboveground P‐use strategies remains unclear. Supporting this possibility, genotypic variation in chickpea has revealed a positive association between early‐stage carboxylates recovered from the rhizosheath and PRE (Feng et al. 2026), suggesting a potential link between root P acquisition and internal P conservation. One possible explanation is that enhanced carboxylate‐mediated P acquisition increases leaf P concentration, thereby providing a larger P pool for subsequent resorption. However, the processes underlying this association remain unclear. Together, these findings suggest that root P acquisition and leaf P resorption are coordinated, yet this coordination has not been directly investigated across developmental stages.

In this study, we investigated variation in PRE across chickpea developmental stages. Although previous studies have examined root P acquisition (Pang et al. 2022; Wouterlood et al. 2004) or have been largely restricted to vegetative stages (Feng et al. 2026) when assessing P‐use traits, to our knowledge no study has simultaneously investigated the coordination between leaf P resorption and root P acquisition within a single experiment across developmental stages. We therefore tested the following two hypotheses under moderate P deficiency: (1) a trade‐off exists between leaf PPUE and PRE; and (2) leaf PRE and PPUE increase during the reproductive stage relative to the vegetative stage, coordinated with a concomitant increase in root P acquisition.

## 2 Materials and Methods

### 2.1 Plant Material and Growth Conditions

Five chickpea accessions (two with high PRE and three with low PRE) were selected from a larger panel of 266 accessions previously screened for PRE variation under moderate P deficiency (Feng et al. 2026). Supporting Information S1: Table S1 provides detailed information on these accessions. Plants were grown in a temperature‐controlled glasshouse at The University of Western Australia (Perth, 31°58′ S, 115°49′ E) from May to December 2024, with mean day/night temperatures of 22/15°C and 75% relative humidity. Plants were grown in 2 L black pots containing 2.5 kg of a soil–sand mix (1:4, w/w). The field soil (0–0.15 m depth, collected from Cunderdin) contained 20 µg P g^−1^ plant‐available P and was mixed with washed river sand. The soil mixture contained 8 mg N kg^–1^ nitrate‐N, 1.7 mg N kg^–1^ ammonium‐N, 10 mg P kg^–1^ Colwell P, 6.4 mg S kg^–1^ sulphur, 135 mg K kg^–1^ Colwell K, 9 mg kg^–1^ organic carbon, and had a pH (CaCl_2_) of 7.2 (Feng et al. 2026). Total P was adjusted to 25 mg P kg^–1^ by adding KH_2_PO_4_. KH_2_PO_4_ was selected as the P source due to its high solubility and its widespread use for precise adjustment of soil P levels in pot experiments. The target soil P concentration was chosen to represent a moderately P‐limiting condition for chickpea. Previous studies have shown that this level is sufficient to induce measurable variation in P acquisition and P‐use traits, while still supporting adequate plant growth and reproductive development (Pang et al. 2022; Pang et al. 2023; Wen et al. 2023). Basal nutrients were supplied before sowing, including 15 mg N kg^–1^ soil as Ca(NO_3_)_2_·4H_2_O, 3.75 mg N kg^–1^ soil as NH_4_Cl, 50 mg S kg^–1^ soil as K_2_SO_4_, 0.12 mg B kg^–1^ soil as H_3_BO_3_, 2 mg Zn kg^–1^ soil as ZnSO_4_·7H_2_O, 0.5 mg Cu kg^–1^ soil as CuSO_4_·5H_2_O, 0.4 mg Mo kg^–1^ soil as Na_2_MoO_4_·2H_2_O and 5 mg Fe kg^−1^ soil as FeNaEDTA. An additional 5 mg N kg^−1^ was applied 1 month after sowing (Feng et al. 2026; Wen et al. 2023). Seeds were surface‐treated with a fungicide, sown (two per pot at 2.5 cm depth), and thinned to one seedling 15 days later. A peat‐based rhizobium inoculant (P Pickel‐T, 360 g L^−1^ thiram, 200 g L^−1^ thiabendazole) was applied post‐emergence. Soil water content was maintained gravimetrically at 80% of pot capacity during vegetative growth and reduced to 70% after pod set. Pots were rearranged regularly within the greenhouse to minimise positional effects, and the soil surface was covered with plastic beads to reduce evaporation. A total of 240 pots (48 per accession) were used. Individual pots were not treated as independent biological replicates. Instead, due to the limited biomass available from individual plants, three pots were pooled to generate a single composite sample for the determination of leaf P fractions and acid phosphatase activity. Each composite sample was considered one biological replicate. For each accession, four independent biological replicates were used for statistical analyses, and additional pots were maintained as reserves or used for destructive sampling at different stages.

All measurements described below were conducted at two developmental stages: the vegetative stage (6 weeks after sowing) and the reproductive stage (12 weeks after sowing, early podding stage).

### 2.2 Photosynthetic Traits and Chlorophyll Determination

Photosynthetic measurements were performed on newly fully expanded leaves of the main stem. Leaves were tagged at full expansion of the 13th leaf to aid identification, and gas exchange was measured at full expansion of the 15th leaf using a LI‐6800 Portable Photosynthesis System (LI‐COR, Lincoln, NE, USA) between 09:30 and 12:00. During measurements, chamber conditions were set to 400 μmol mol^−1^ CO_2_, 1500 μmol photons m^–2^ s^–1^ PAR supplied by an R90 LED light source (90% red light), ~ 65% relative humidity, and 25°C. Air flow was maintained at 500 μmol s^−1^ with a mixing fan speed of 10 000 rpm to ensure uniform mixing of CO_2_ and water vapour.

The 15th leaf and one adjacent leaf below (14th leaf) were harvested pre‐dawn on the day following gas exchange measurements, placed in an ice box, transported to the laboratory, and weighed for fresh weight. Leaves were then immediately scanned for leaf area using a flatbed scanner at 300 dpi (Epson Perfection v850 pro, Tokyo, Japan), followed by analysis using WinRhizo 2009 software (Regent Instruments Inc., Quebec, Canada). Subsequently, leaves were oven‐dried at 60°C for 48 h to determine dry weight. Leaf mass per area (LMA) was calculated as the ratio of leaf dry weight to leaf area, while leaf dry matter content (LDMC) was calculated as the ratio of leaf dry weight to fresh weight. Leaf thickness was estimated by dividing LMA by LDMC (Vile et al. 2005). Mass‐based photosynthetic rate (A_mass_) was calculated by dividing area‐based photosynthetic rate (A_area_) by LMA. Leaf instantaneous intrinsic water‐use efficiency (WUE_i_) was determined as the ratio of A_area_ to stomatal conductance (g_sw_).

Three to four leaflets from the 15th leaf were collected into plastic bags, placed on ice, and weighed immediately upon return to the laboratory. The samples were then immersed in 10 mL of 80% (v/v) acetone and wrapped in aluminium foil for 48 h to extract chlorophyll and carotenoids. Absorbance at 663, 645 and 470 nm was measured using a spectrophotometer (AQ8100, Thermo Fisher Scientific, Waltham, MA, USA), and pigment concentrations were calculated according to Chazaux et al. (2022).

### 2.3 Elemental Analyses and Phosphorus Fractions

The 14th and 15th leaves from plants similar to those used for photosynthesis measurement were rapidly harvested, wrapped in aluminium foil, immediately flash‐frozen in liquid nitrogen, and stored at –80°C. To ensure sufficient material for analysis, three individuals were pooled to form one biological replicate. A portion of the samples was freeze‐dried (6 days, Eyela FDU‐2100, Tokyo Rikakikai Co. Ltd, Tokyo, Japan) and ground into a fine powder using a Geno/Grinder 2000 (Spex SamplePrep, Metuchen, NJ, USA). One subsample (~ 50 mg) was acid‐digested (HNO_3_: HClO_4_, 3:1, v/v) and analysed using inductively coupled plasma optical emission spectroscopy (ICP‐OES) to determine leaf concentrations of P, potassium (K), calcium (Ca), iron (Fe), manganese (Mn), and zinc (Zn). A second subsample (~ 100 mg) was used to quantify P fractions following Hayes et al. (2022). In brief, samples were sequentially extracted using chloroform:methanol:formic acid (12:6:1, v/v/v) and chloroform:methanol:water (1:2:0.8, v/v/v) to separate lipid P from water‐soluble P fractions. The aqueous phase contained inorganic P and metabolite P, with inorganic P quantified by acetic acid extraction and metabolite P calculated by difference. The remaining pellet was subsequently extracted with 2.5% (v/v) trichloroacetic acid at ~95°C to obtain nucleic acid P, while the final insoluble residue was designated residual P. All fractions were acid‐digested with HNO_3_–HClO_4_ (3:1 v/v) and analysed for P using the malachite green colorimetric method (Motomizu et al. 1983). Approximately 25 mg of a third subsample was used to determine leaf N concentration using a Vario Macro combustion analyser (Elementar Analysensysteme GmbH, Hanau, Germany).

### 2.4 Leaf Protein Extraction and Acid Phosphatase Activity

Leaf proteins and acid phosphatase activity were measured following Knowles and Plaxton (2013), with minor modifications. Frozen samples stored at −80°C were transferred to liquid nitrogen, then quickly ground into a fine powder in a pre‐chilled mortar. Liquid nitrogen was added as needed to maintain the frozen state during grinding. After grinding, ~ 0.5 g of the powdered sample was weighed into microtubes and kept on ice. Then, 1.5 mL of extraction buffer was added, and the mixture was gently mixed to ensure uniform contact between the buffer and the sample. The mixture was centrifuged at 11 000 *g* for 5 min at 4°C, and the supernatant was collected. Acid phosphatase activity was measured immediately at room temperature (24°C) over a 10‐min period, and absorbance was recorded at 410 nm (Tabatabai 1994). Aliquots of the extract were used for protein quantification using Brilliant Blue dye. Protein concentration was determined using the Bradford assay, with bovine serum albumin (BSA, 2.0 mg mL^–1^) as the standard, and absorbance was measured at 595 nm.

### 2.5 Soil Acid Phosphatases Activity and Rhizosheath Carboxylates

At harvest, the entire root system was carefully collected. The plant was held at the base of the main stem, and excess soil was gently shaken off, while the soil remaining tightly adhered to the root surface was defined as rhizosheath soil (Pang et al. 2017).

Approximately 1 g of rhizosheath soil was collected to determine soil acid phosphatase activity following Tabatabai (1994). In brief, rhizosheath soil was weighed into 10 mL plastic tubes and incubated with *p*‐nitrophenyl phosphate in modified universal buffer (pH 6.5) at 37°C for 1 h. The reaction was terminated by adding 1 mL 0.5 M CaCl_2_ and 4 mL 0.5 M NaOH, and the released *p*‐nitrophenol was quantified spectrophotometrically at 420 nm. Subsequently, the entire root system was immersed in 100 mL of 0.2 mM CaCl_2_ to preserve cell integrity, and gently agitated to detach the rhizosheath soil. The pH of the resulting rhizosheath slurry was measured using a portable pH metre. A subsample (~ 10 mL) of carboxylate extract recovered from the rhizosheath soil was then filtered through a 0.45‐µm syringe filter. A 1‐mL aliquot of the filtrate was transferred to an HPLC vial, acidified with 20 µL orthophosphoric acid and stored at −20°C until analysis. Carboxylates were subsequently quantified by high‐performance liquid chromatography (HPLC) as described by Cawthray (2003). Briefly, carboxylates were analysed using reversed‐phase HPLC (Waters 600E pump and 717 autosampler; Waters, Milford, MA, USA) equipped with a C18 column. Separation was achieved using a mobile phase consisting of 93% (v/v) 25 mM KH_2_PO_4_ (pH 2.5) and 7% (v/v) methanol at a flow rate of 1.0 mL min^−1^, with detection at 210 nm. Organic acids (malic, malonic, lactic, acetic, maleic, citric, succinic, *cis*‐aconitic, *trans*‐aconitic, and fumaric acids) were identified by retention time and quantified using external calibration curves prepared from authentic standards following Cawthray (2003). After extraction, the roots were rinsed with deionised water. Plants were then separated into roots, stems and leaves, and oven‐dried at 70°C for 72 h to determine dry biomass.

### 2.6 Nutrient‐Resorption Efficiency

Fully expanded leaves were tagged using small plastic labels at two developmental stages – 6 weeks after sowing (vegetative stage) and 12 weeks after sowing (reproductive stage: early podding). Once the tagged leaves and their adjacent leaves reached full senescence, leaves from the corresponding positions were collected as senesced leaf samples.

Leaf N‐ and P‐resorption efficiencies were calculated as:

$$\text{PRE}(\%)=(P_{g}-P_{s})/P_{g}\times 100$$

$$\text{NRE}(\%)=(N_{g}-N_{s})/N_{g}\times 100$$

where *P_g_* and *N_g_* represent the concentrations of P and N in green leaves, and *P_s_* and *N_s_* represent those in senesced leaves.

### 2.7 Statistical Analyses

Trait differences among accessions at the same developmental stage (e.g., leaf nutrient concentrations and rhizosheath carboxylates) were analysed using one‐way analysis of variance (ANOVA), followed by Tukey's HSD test for post hoc comparisons. Log transformations were applied where necessary to ensure normality, and homogeneity of variance was assessed using Bartlett's test. To assess the overall effect of growth stage on key traits across accessions, linear mixed‐effects models were fitted using the **lme4** and **lmerTest** packages. Growth stage was included as a fixed effect, and accession was included as a random effect. The full model was compared with a null model (intercept‐only) using likelihood‐ratio tests and the Akaike Information Criterion (AIC). Following the mixed‐effects analysis, differences between vegetative and reproductive stages within individual accessions were evaluated using Welch's *t*‐test. Principal component analysis (PCA) was conducted using the ‘prcomp' function to identify variation in key leaf traits between stages, with data scaled prior to analysis. Horn's parallel analysis was subsequently performed using the ‘paran' function to determine the number of component dimensions to retain. All statistical analyses and figures were generated using R 4.3.0 (R Core Team, 2023).

## 3 Results

### 3.1 Variation in Phenology and Biomass Allocation

Biomass allocation varied significantly among chickpea accessions and between vegetative and reproductive stages (Figure 1). During the vegetative stage, biomass was predominantly allocated to leaves and roots, followed by stems. We observed no significant differences in leaf mass ratio among the five accessions (*F*_*4,20*_ = 1.64, *p* = 0.20). Similarly, stem and root allocation was generally consistent among accessions, except for ICC9590, which allocated significantly less biomass to roots and more to stems than the other accessions did (*p* < 0.05, Tukey's HSD).

**Figure 1:**
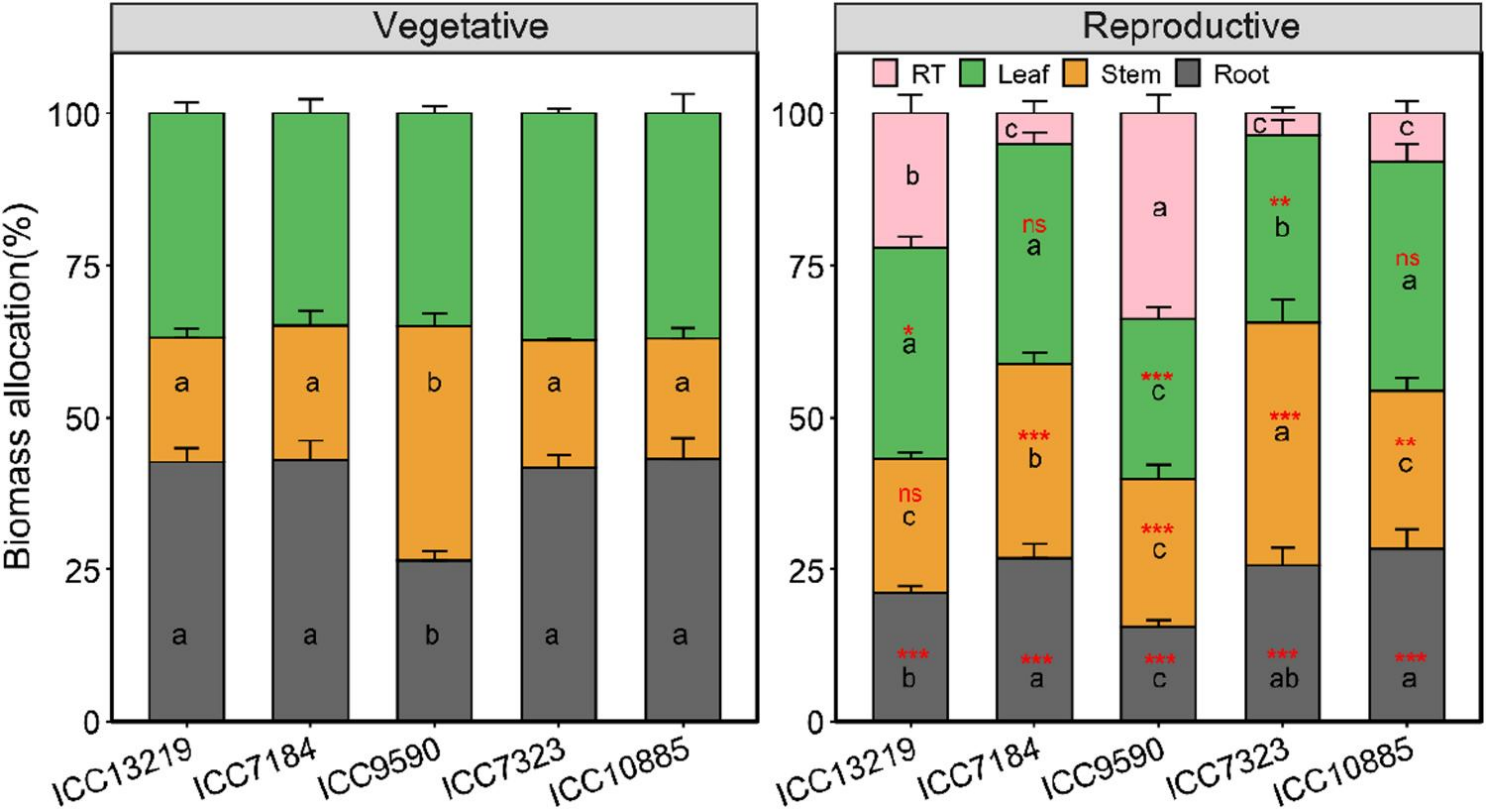
Biomass allocation in five chickpea accessions at the vegetative (6 weeks after sowing) and reproductive stages (12 weeks after sowing) under moderate P deficiency (25 mg P kg^−1^). RT represents reproductive tissues, including flowers, pods and seeds. Data are means ± SE (*n* = 5). Differences among accessions within the same organ are indicated by lowercase (*p* < 0.05, Tukey's HSD). Asterisks denote differences between growth stages for the same organ: ns: not significant, *p < 0.05, **p < 0.01, ***p < 0.001 (Welch's *t*‐test).

The five accessions differed in phenology, with ICC9590, ICC10885 and ICC13219 reaching flowering and podding earlier than the others (Supporting Information S1: Table S2). Biomass allocation patterns shifted markedly at the reproductive stage (Figure 1). All accessions increased investment in reproductive tissues (RT), with ICC13219 and ICC9590 allocating significantly more to RT than the other accessions did. Root biomass allocation decreased in all accessions compared with the vegetative stage, with ICC9590 and ICC13219 showing the most pronounced reductions (*p* < 0.001, *t*‐test).

Leaf allocation during the reproductive stage was relatively stable in ICC7184 and ICC10885, but decreased significantly in ICC7323 and ICC9590 (*p* < 0.01 or *p* < 0.001). In contrast, stem allocation increased significantly in ICC7323, ICC7184 and ICC10885, whereas it decreased in ICC9590 (Figure 1).

### 3.2 Photosynthetic Traits

Area‐based photosynthetic rate (A_area_) increased significantly from the vegetative to the reproductive stage in all five accessions (Figure 2), with an average increase of 36%, ranging from 26% in ICC9590 to 40% in ICC7184. However, the increase in ICC10885 was not statistically significant (*t* = –1.87, df = 5.4, *p* = 0.12). ICC7184 exhibited the highest A_area_ values at both stages, while ICC10885 had the lowest, although differences among accessions were not significant (*F*_*4,20*_ = 1.76, *p* = 0.18). Similarly, we observed no significant genotypic differences in A_area_ at the reproductive stage (*F*_*4,20*_ = 2.16, *p* = 0.11). In contrast, A_mass_ was stable, showing no significant differences either between stages or among accessions (Figure 2b).

**Figure 2:**
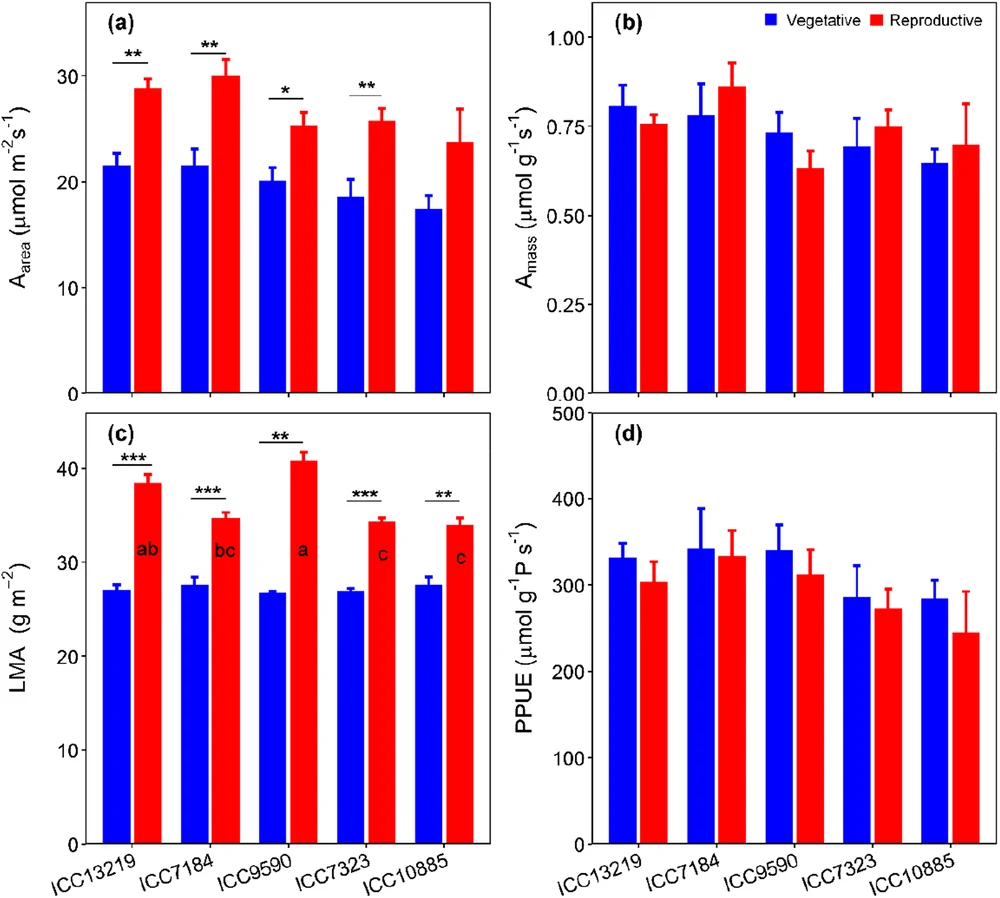
Leaf photosynthetic traits at the vegetative (blue, 6 weeks after sowing) and reproductive stage (red, 12 weeks after sowing) in five chickpea accessions under moderate P deficiency (25 mg P kg^−1^): (a) area‐based photosynthesis (A_area_) and (b) mass‐based photosynthesis (A_mass_), (c) leaf mass per area (LMAc), and (d) photosynthetic phosphorus‐use efficiency (PPUE). Data are means ± SE (*n* = 4). Differences among accessions within the same growth stage are indicated by lowercase letters (*p* < 0.05, Tukey's HSD). Asterisks denote differences between growth stages for the same accession: *p < 0.05, **p < 0.01, ***p < 0.001 (Welch's *t*‐test). Non‐significant differences are not shown. [Color figure can be viewed at wileyonlinelibrary.com]

Leaf mass per area significantly increased across all accessions from the vegetative to the reproductive stage (average increase 30%; Figure 2c), with the most pronounced increases in ICC13219 (42%) and ICC9590 (53%) (*p* < 0.001 and *p* < 0.01, respectively). At the reproductive stage, LMA differed significantly among accessions (*p* < 0.05, Tukey's HSD), with ICC9590 exhibiting the highest values and ICC10885 the lowest.

Photosynthetic P‐use efficiency did not vary significantly between stages or among accessions (Figure 2d), although slight genotypic differences were apparent. ICC7184 and ICC13219 tended to maintain higher PPUE values, whereas ICC7323 and ICC10885 exhibited the lowest.

### 3.3 Leaf Nutrient Concentration and Nutrient Resorption

Leaf P concentrations and PRE varied significantly between growth stages under moderate P deficiency (Figure 3). At the vegetative stage, green leaf P concentration (P_g_) ranged from 2.16 mg P g^−1^ in ICC9590 to 2.44 mg P g^–1^ in ICC7323, with no significant differences among accessions (*F*_*4,15*_ = 2.09, *p* = 0.13).

**Figure 3:**
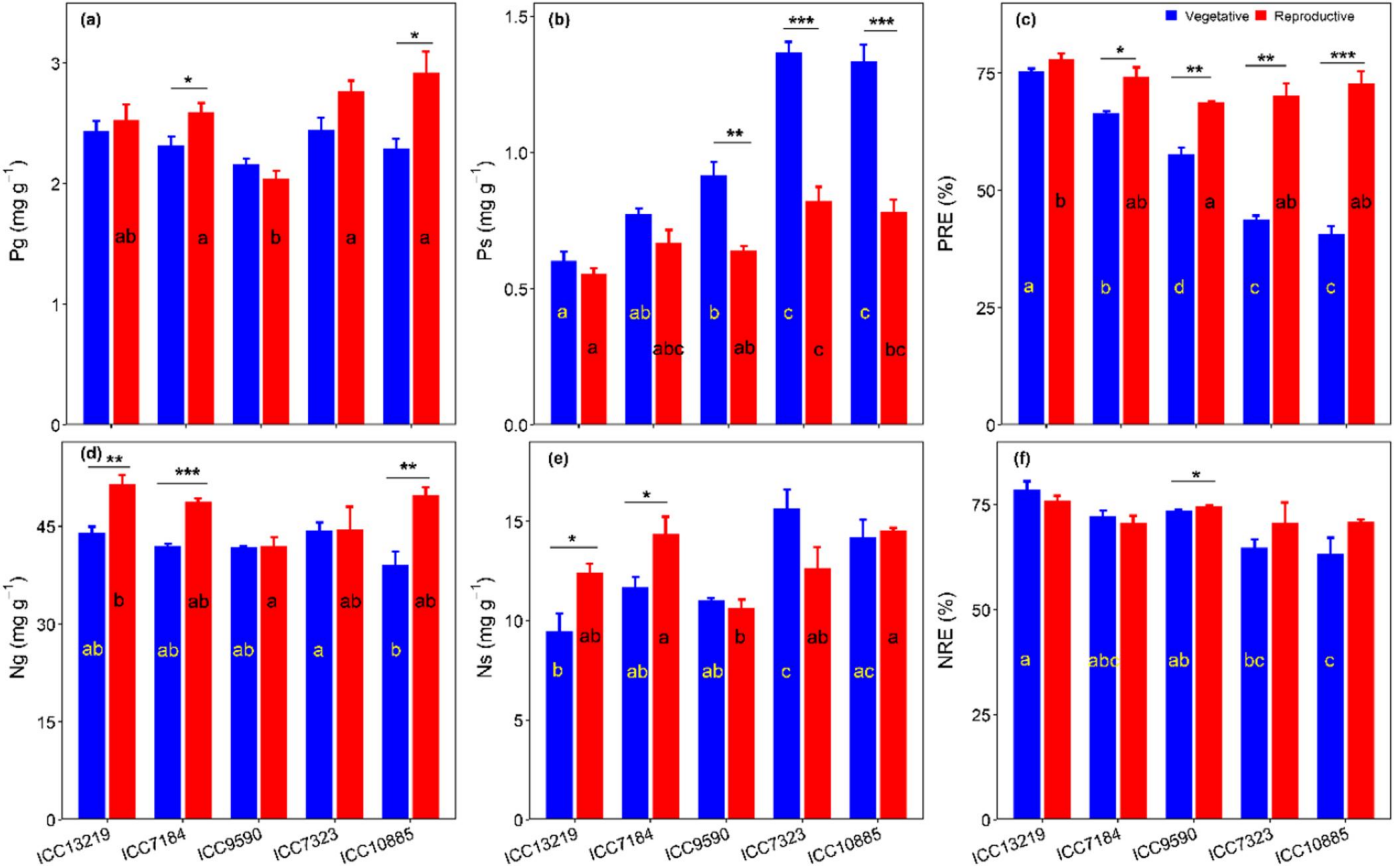
Leaf nutrient concentrations and resorption efficiency at the vegetative (blue, 6 weeks after sowing) and reproductive stage (red, 12 weeks after sowing) in five chickpea accessions under moderate P deficiency (25 mg P kg^−1^): (a) green leaf phosphorus (P_g_), (b) senesced leaf phosphorus (P_s_), (c) phosphorus‐resorption efficiency (PRE), (d) green leaf nitrogen (N_g_), (e) senseced leaf nitrogen (N_s_), and (f) nitrogen‐resorption efficiency (NRE). Data are means ± SE (*n* = 4). Differences among accessions within the same growth stage are indicated by lowercase letters (*p* < 0.05, Tukey's HSD). Asterisks denote differences between growth stages for the same accession: *p* < 0.05, **p < 0.01, ***p < 0.001 (Welch's *t*‐test). Non‐significant differences are not shown. [Color figure can be viewed at wileyonlinelibrary.com]

Compared with the vegetative stage, P_g_ increased significantly during the reproductive stage in ICC10885 and ICC7184 (*χ^2^* = 9.6, *df* = 1, *p* = 0.002), but it did not change significantly in ICC13219, ICC9590 and ICC7323. At the reproductive stage, we observed significant genotypic differences in P_g_, with ICC7184, ICC10885 and ICC7323 exhibiting higher concentrations than ICC9590 did.

Senesced leaf P (P_s_) differed significantly among accessions at both stages, with pronounced differences at the vegetative stage (Figure 3b). ICC13219 and ICC7184 had the lowest P_s_, whereas ICC7323 and ICC10885 had the highest, more than twice that of ICC13219. At the reproductive stage, ICC13219 still had the lowest P_s_, significantly lower than ICC7323 and ICC10885, although the differences were less pronounced. Overall, P_s_ decreased from the vegetative to the reproductive stage in all accessions, including ICC13219.

Phosphorus‐resorption efficiency increased from the vegetative to the reproductive stage in all accessions (Figure 3c), with ICC7323 and ICC10885 showing the greatest increases (26% and 32%, respectively). ICC13219 consistently exhibited the highest PRE, indicating the most efficient P remobilisation.

Green leaf N (N_g_) increased during the reproductive stage in ICC13219, ICC7184 and ICC10885 (Figure 3d), with ICC13219 showing the highest N_g_ overall. In contrast, senesced leaf N (N_s_) did not follow a consistent trend across stages. It increased in ICC13219 and ICC7184 but decreased non‐significantly in ICC7323 and ICC9590 (Figure 3e).

Nitrogen‐resorption efficiency (NRE) varied little among accessions (Figure 3f). ICC13219 maintained the highest NRE at both stages, whereas ICC10885 had the lowest. ICC9590 showed an increase in NRE from the vegetative to the reproductive stage, while the NRE of other accessions was largely unchanged. We observed no significant genotypic differences in NRE at the reproductive stage. Unlike PRE, NRE did not increase significantly at the reproductive stage (*χ^2^* = 1.8, *df* = 1, *p* = 0.18).

### 3.4 Phosphorus Fractions

Green leaves at the reproductive stage exhibited increased concentrations of various P fractions compared with the vegetative stage. Specifically, nucleic acid P increased significantly in ICC10885, while lipid P significantly increased in ICC7323 and ICC10885 (Figure 4). In senesced leaves, stage‐dependent differences were also evident, with inorganic P declining substantially during the reproductive stage. Resorption efficiency varied among P fractions, with nucleic acid P showing the highest mean efficiency (85%), while inorganic P and metabolic P showed the lowest (44% and 31%, respectively).

**Figure 4:**
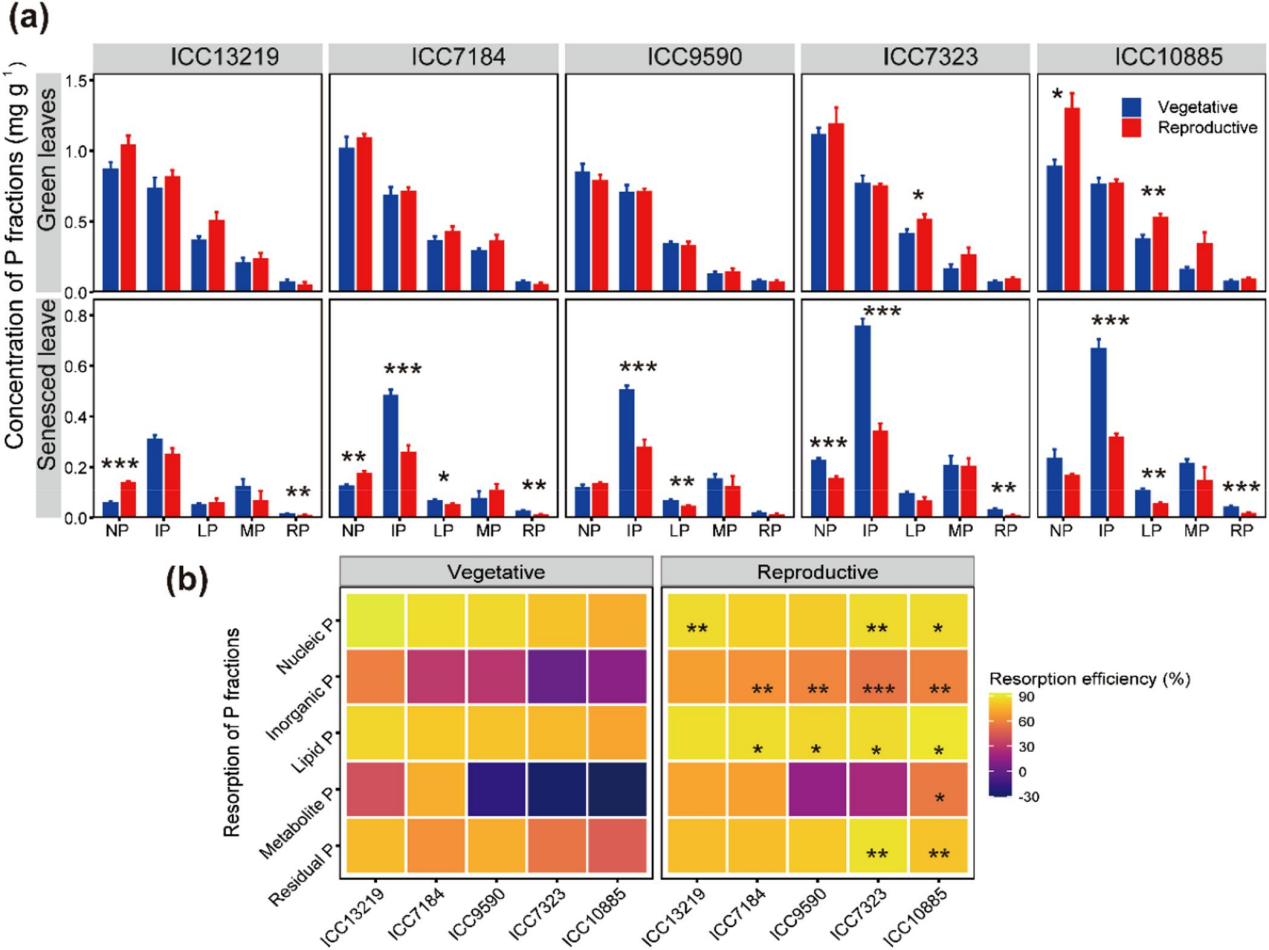
(a) Concentration of five phosphorus (P) fractions in green (top panels) and senesced (bottom panels) leaves at the vegetative (six weeks after sowing) and reproductive stage (12 weeks after sowing) in five chickpea accessions, and (b) resorption efficiency of each fraction. Data are means ± SE (*n* = 4). Colours in (b) represent bootstrap means from 10 000 resamples. Asterisks denote differences between growth stages for the same P fraction within each accession: *p < 0.05, **p < 0.01, ***p < 0.001, Welch's *t*‐test). NP, IP, LP, MP, and RP represent nucleic acid P, inorganic P, lipid P, metabolite P and residual P, respectively. [Color figure can be viewed at wileyonlinelibrary.com]

During the vegetative stage, low‐PRE accessions had particularly low resorption efficiency for inorganic and metabolic P, sometimes showing negative values, indicating P concentrations in senesced leaves exceeded those in green leaves. Resorption efficiency generally increased during the reproductive stage, with the largest increases observed for inorganic P (Figure 4; S2).

### 3.5 Leaf Thickness, Chlorophyll Concentration, Acid Phosphatase Activity and Lifespan

Leaf thickness increased significantly from the vegetative to the reproductive stage across all accessions (average increase of 22%, Figure 5). We observed the largest increase in ICC13219 (*p* < 0.001), while ICC7184 showed minimal change (*p* = 0.09). At the reproductive stage, ICC13219 had significantly thicker leaves, whereas ICC7184 and ICC7323 had the thinnest (Figure 5a).

**Figure 5:**
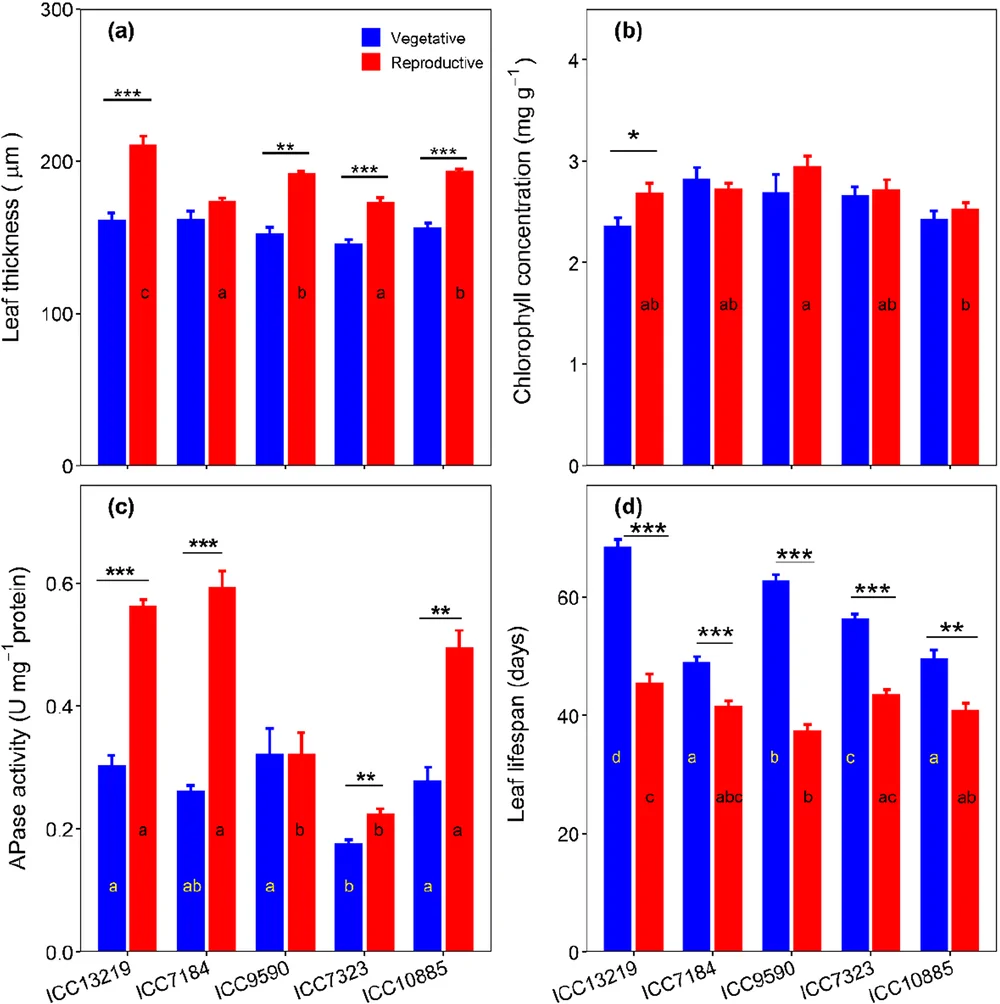
Leaf morphology and physiology at the vegetative (blue, 6 weeks after sowing) and reproductive stage (red, 12 weeks after sowing) in five chickpea accessions under moderate P deficiency (25 mg P kg^−1^): (a) leaf thickness, (b) chlorophyll concentration, (c) leaf acid phosphatase activity, and (d) leaf lifespan. Data are means ± SE (*n* = 4). Differences among accessions within the same growth stage are indicated by lowercase letters (*p* < 0.05, Tukey's HSD). Asterisks denote differences between two growth stages for the same accession: *p < 0.05, **p < 0.01, ***p < 0.001 (Welch's *t*‐test). Non‐significant differences are not shown. [Color figure can be viewed at wileyonlinelibrary.com]

Chlorophyll concentration varied moderately among accessions and stages (Figure 5b). A slight but significant average increase at the reproductive stage (Table S3, *p* = 0.02) was mainly driven by ICC13219.

Leaf acid phosphatase (APase, Table S3) activity increased strongly from the vegetative to the reproductive stage (Figure 5c), with significant increases in ICC13219, ICC7184 and ICC10885 (*p* < 0.001 or *p* < 0.01). ICC7184 showed the highest APase activity at the reproductive stage, while ICC7323 and ICC9590 maintained relatively low activities (*p* < 0.05).

Leaf lifespan declined markedly from the vegetative to the reproductive stage (average reduction of 27%, Figure 5d). ICC13219 had the longest lifespan at both stages, whereas ICC7184 (vegetative stage) and ICC9590 (reproductive stage) had the shortest.

Carboxylate recovered from the rhizosheath varied significantly among accessions and stages (Figure 6). On a root dry weight basis, exudation increased significantly at the reproductive stage (*p* < 0.05, Figure 6a), with ICC10885 and ICC13219 showing the highest values, and ICC9590 the lowest. Normalised per plant, the reproductive stage showed greater increases, particularly in ICC7184 and ICC10885 (Figure 6b).

**Figure 6:**
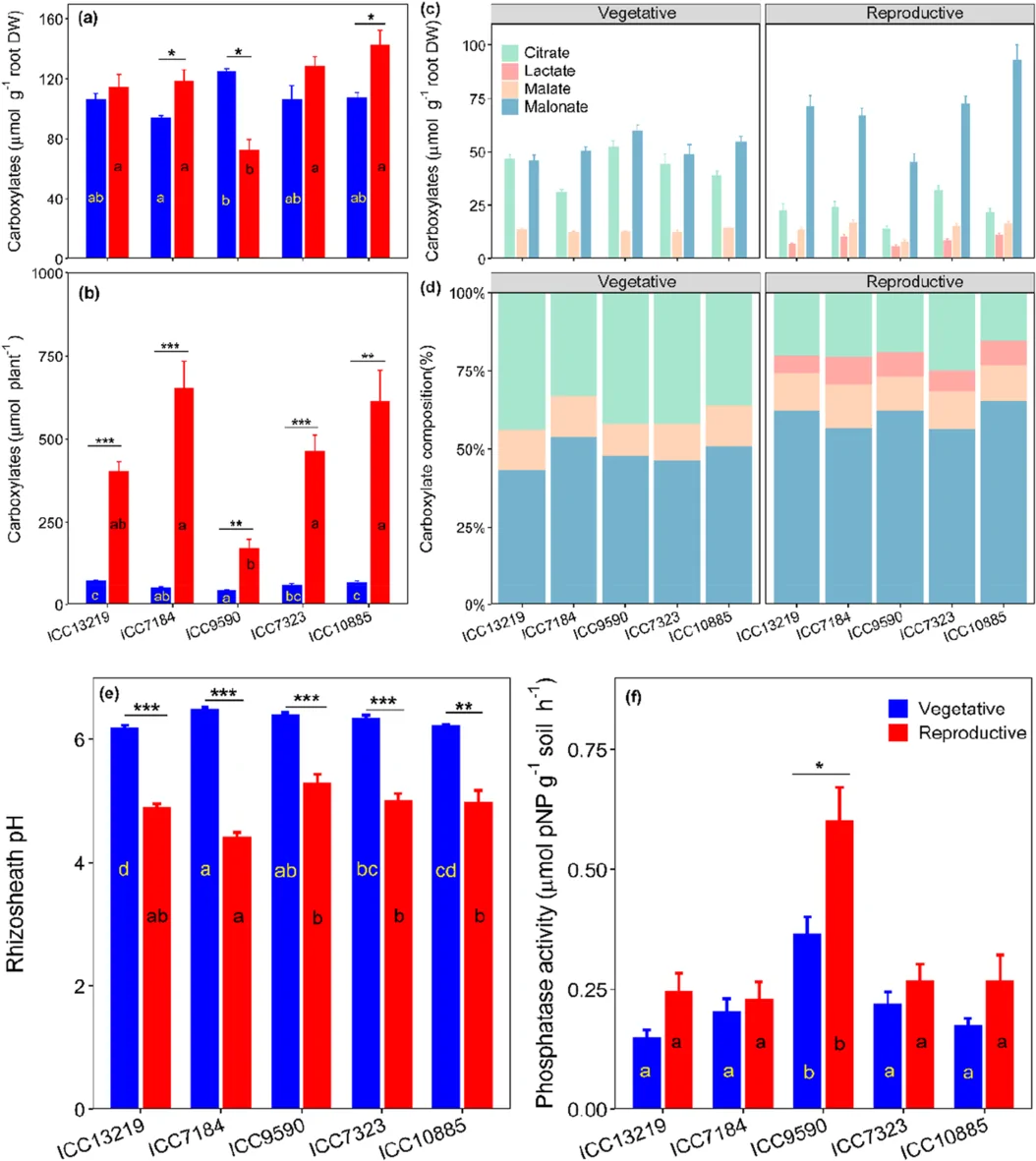
Carboxylates recovered from the rhizosheath soil and other rhizosphere traits at the vegetative (blue, 6 weeks after sowing) and reproductive stage (red, 12 weeks after sowing) in five chickpea accessions under moderate P deficiency (25 mg P kg^−1^): (a) carboxylates per root dry weight, (b) carboxylates per plant, (c) composition of exuded carboxylates, (d) proportional allocation of carboxylates, (e) rhizosheath pH, and (f) soil phosphatase activity. Data are means ± SE (n ≥ 4). Differences among accessions within the same growth stage are indicated by lowercase letters (*p* < 0.05, Tukey's HSD). Asterisks denote differences between growth stages for the same accession: *p < 0.05, **p < 0.01, ***p < 0.001 (Welch's *t*‐test). Non‐significant differences are not shown. [Color figure can be viewed at wileyonlinelibrary.com]

Citrate and malonate were the predominant carboxylates, together accounting for over 70% of total carboxylates (Figure 6c,d). During the reproductive stage, the amount of malonate increased slightly, that of citrate decreased significantly, and that of malate remained low. Lactate was detected only at the reproductive stage, comprising ~10% of total carboxylates.

Rhizosheath pH decreased significantly at the reproductive stage in all accessions (Figure 6e). Rhizosheath soil phosphatase activity exhibited genotypic and stage‐dependent differences (Figure 6f), with only ICC9590 showing a significant increase (*p* < 0.05); other accessions showed non‐significant upward trends.

### 3.6 Principal Component Analysis

The PCA of 24 leaf traits across five accessions explained 57.2% of total variation (Figure 7; Table 1), with PC1 and PC2 accounting for 38.7% and 18.5%, respectively. PC1 was primarily associated with leaf gas exchange and functional traits, with high positive loadings (≥ 0.25) for transpiration rate (E, 0.30), g_sw_ (0.28), LMA (0.26), leaf thickness (0.27) and PRE (0.27). PC2 was dominated by traits related to photosynthetic P‐ and N‐use efficiency, with high loadings for P_g_ (0.35), K (0.33) and N_s_ (0.28), and negative loadings for PPUE (− 0.40) and PNUE (− 0.32).

**Figure 7:**
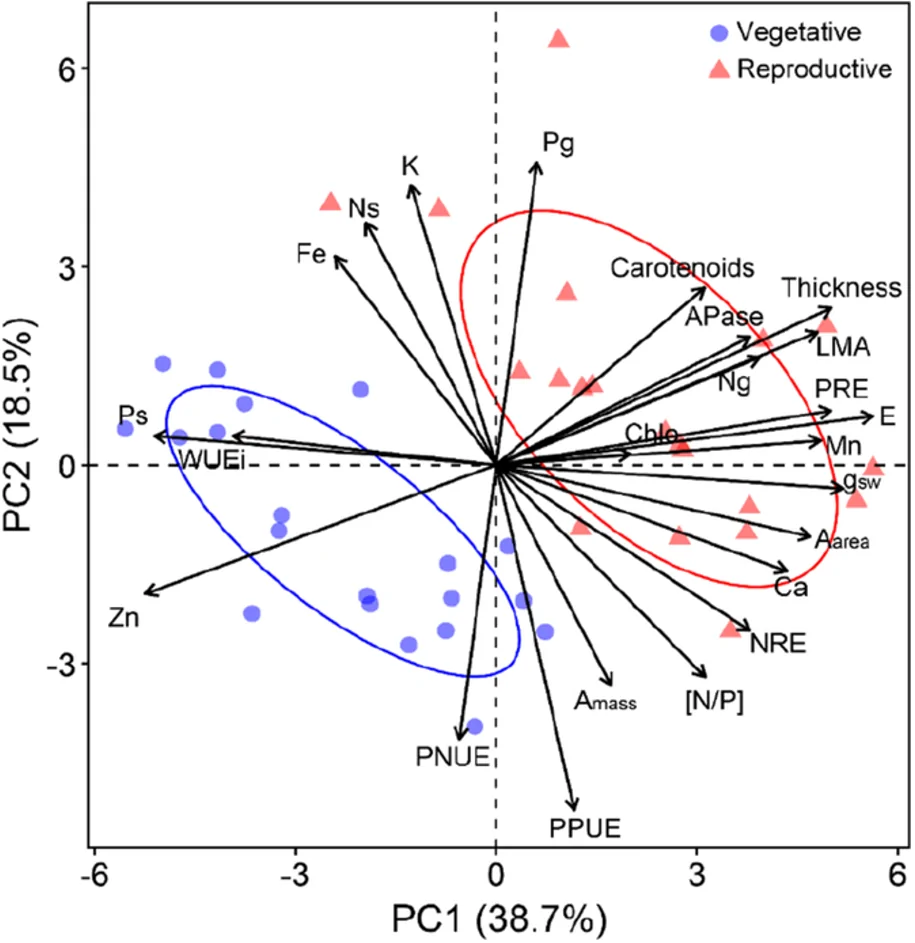
Principal component analysis of 24 leaf traits for the vegetative (blue, 6 weeks after sowing) and reproductive stage (red, 12 weeks after sowing) in 5 chickpea accessions under moderate P deficiency (25 mg P kg^–1^). APase, leaf acid phosphatase activity; LMA, leaf mass per area; NRE/PRE, nitrogen/phosphorus‐resorption efficiency; PNUE/PPUE, photosynthetic nitrogen/phosphorus‐use efficiency; E, transpiration rate; g_sw_ stomotal conductance; A_area_/A_mass,_ photosynthetic rate based on area or mass; WUE_i_, intrinsic water‐use efficiency; Chlo, chlorophyll concentration; Ca, K, Fe, Zn, Mn, leaf calcium, potassium, iron, zinc, and manganese concentrations. [Color figure can be viewed at wileyonlinelibrary.com]

**Table 1:** Principal component loadings and variance explained by the first three principal components derived from 24 leaf traits across five chickpea accessions under moderate phosphorus deficiency (25 mg P kg^–1^).

Variable	PC1	PC2	PC3
APase	0.2	0.15	–0.19
Carotenoids	0.17	0.21	0.03
Chlorophyll	0.11	0.01	0.24
Thickness	**0.27**	0.18	0.05
Transpiration rate	**0.3**	0.06	–0.05
A_area_	**0.25**	–0.08	–**0.31**
g_sw_	**0.28**	–0.03	–0.03
WUE_i_	–0.21	0.03	–0.14
LMA	**0.26**	0.16	0.12
A_mass_	0.09	–0.26	–**0.48**
P_g_	0.03	**0.35**	**−0.26**
PPUE	0.06	–**0.4**	–**0.26**
PNUE	−0.03	**−0.32**	−0.4
P_s_	**−0.27**	0.03	0.01
N_g_	0.21	0.13	−0.15
[N/P]	0.17	–**0.25**	0.16
N_s_	–0.1	**0.28**	−0.22
NRE	0.2	–0.19	0.12
PRE	**0.27**	0.06	–0.09
Ca	0.23	–0.12	0.13
Fe	–0.13	0.24	–0.16
K	–0.07	**0.33**	**−0.27**
Mn	**0.26**	0.03	0.02
Zn	–**0.28**	–0.15	0
Variability (%)	39	19	10
Cumulative variability (%)	39	57	68

*Note:* The highest variable loading scores (≥ 0.25) in the first three principal components are highlighted in bold.

Abbreviations: A_area_/A_mass,_ photosynthetic rate based on area or mass; APase, leaf acid phosphatase activity; Ca, K, Fe, Zn, Mn, leaf calcium, potassium, iron, zinc, and manganese concentrations; Carotenoids/Chlorophyll, leaf carotenoids/chlorophyll concentration; Chlo, chlorophyll concentration; g_sw,_ stomatal conductance; LMA, leaf mass per area; NRE/PRE, nitrogen/phosphorus‐resorption efficiency; PNUE/PPUE, photosynthetic nitrogen/phosphorus use‐efficiency; Thickness, estimated leaf thickness; WUE_i_, intrinsic water‐use efficiency.

The PCA biplot clearly separated the vegetative (blue circles) and reproductive (red triangles) stages along PC1. Vegetative stage samples clustered on the negative PC1 side, associated with P_s_, WUE_i_ and leaf [Zn]. Reproductive stage samples clustered on the positive PC1 side, characterised by higher LMA, leaf thickness, PRE, g_sw_, E and A_area_. Notably, PRE and PPUE were separated along PC1 and PC2, respectively, indicating no trade‐off or coordination between these traits.

## 4 Discussion

Our results demonstrate that chickpea enhances PRE during its reproductive stage, while maintaining relatively stable leaf PPUE, without evidence of a PPUE‐PRE tradeoff. We observed increased acid phosphatase activity and carboxylates recovered from the rhizosheath alongside increased leaf PRE, suggesting a potential coordination between root P acquisition and internal P conservation that may contribute to overall plant P‐use economy. Leaf transpiration was also positively associated with PRE, indicating a possible role in P remobilisation, although the underlying mechanisms remain unclear. These findings advance our understanding of the observed coordination between P‐acquisition and P‐conservation strategies during reproductive development in chickpea.

### 4.1 Changes in Leaf Morphology and Physiology During Reproduction

Plants respond and adjust to environmental variation and developmental progression by altering leaf morphology and physiology (Feng et al. 2022; Feng et al. 2023; Tully et al. 2013). In this study, chickpea LMA increased by an average of 34% during the reproductive stage relative to the vegetative stage (Figure 2c, Table 2), accompanied by a similar 34% increase in A_area_. These changes were primarily associated with increased leaf thickness (22%), rather than tissue density, and A_mass_ did not significantly change (Figure 2b). More broadly, interspecific variation in LMA often is explained by differences in leaf thickness rather than tissue density (Lambers and Poorter 1992). Consistent with this general pattern, fast‐growing mangrove species exhibit higher LMA and leaf thickness without differences in tissue density (Wei et al. 2025).

**Table 2:** Key leaf trait parameters and percentage changes showing significant differences among five accessions across growth stages based on linear mixed‐effects models (means ± SE, *n* = 20).

Variable	Vegetative	Reproductive	Change percentage (%)
APase (U mg^–1^ protein)	0.27 ± 0.01	0.44 ± 0.03	63.5
Carotenoids (mg g^–1^)	0.42 ± 0.01	0.49 ± 0.01	18.0
Chlorophyll (mg g^–1^)	2.60 ± 0.06	2.75 ± 0.05	6.1
Thickness (μm)	155.7 ± 2.1	189.5 ± 3.6	21.7
Transpiration rate (mmol m^–2^ s^–1^)	2.12 ± 0.18	5.17 ± 0.29	143.7
A_area_ (μmol m^–2^ s^–1^)	19.9 ± 0.8	26.8 ± 1.1	34.4
g_sw_ (mol m^–2^ s^–1^)	0.23 ± 0.02	0.41 ± 0.03	73.2
WUE_i_ (μmol mol^–1^)	89.9 ± 4.7	73.2 ± 5.7	−18.6
LMA (g m^–2^)	27.2 ± 0.3	36.4 ± 0.8	33.9
P_g_ (mg g^–1^)	2.33 ± 0.04	2.57 ± 0.08	10.2
P_s_ (mg g^–1^)	1.00 ± 0.07	0.69 ± 0.03	−30.7
N_g_ (mg g^–1^)	42.2 ± 0.07	47.4 ± 0.03	12.1
PRE (%)	56.8 ± 3.1	72.7 ± 1.1	28.1
Zn (mg kg^–1^)	160.1 ± 3.1	113.3 ± 2.5	−29.2

Abbreviations: A_area_, photosynthetic rate based on area; APase, leaf acid phosphatase activity; Carotenoids/Chlorophyll, leaf carotenoids/chlorophyll concentration; g_sw_ stomatal conductance; LMA, leaf mass per area; P_g_, P_s_, N_g_, leaf phosphorus (P) in green leaves, P concentration in senesced leaves, and nitrogen concentration in green leaves; PRE, P resorption efficiency; Thickness, estimated leaf thickness; WUE_i_, intrinsic water‐use efficiency; Zn, leaf zinc concentration.

Nutrient acquisition was also enhanced during reproduction. Increased P acquisition was supported by elevated carboxylates recovered from the rhizosheath and soil phosphatase activity (Figure 6), consistent with previous studies in chickpea (*Cicer arietinum*) (Pang et al. 2022; Wouterlood et al. 2004). Biological nitrogen fixation may also have intensified, given that nodules were sparse during the vegetative stage but abundant during the reproductive stage in this study (Figure S1), coinciding with marked increases in root P (from 1.51 to 2.32 mg P g^–1^) and N (from 15.8 to 32.8 mg N g^–1^) concentrations (Figure S2). These increased nutrient concentrations supported higher leaf photosynthetic pigment concentrations and a greater photosynthetic capacity (Figure 5b, Table 2).

The vegetative stage occurred during the Mediterranean winter in Western Australia (May–July), with cloudy, rainy conditions, whereas the reproductive stage coincided with longer, more intense light periods after August. Increased light availability during the reproductive stage may have contributed to greater leaf thickness, as higher irradiance has been shown to increase LMA, leaf thickness (Hanba et al. 2002; Fan et al. 2013; Kong et al. 2016), and stomatal size and density (Franks and Farquhar 2001; Lawson and Blatt 2014). Increased stomatal density may increase stomatal conductance, which is associated with elevated transpiration rates and reduced water‐use efficiency (Table 2). The observed increase in A_area_ may have been associated with the faster transpiration rates. One possible explanation is that faster transpiration rates might enhance xylem delivery of cytokinins which has been implicated in photosynthetic acclimation to increased irradiance in tobacco (*Nicotiana tabacum*) and Arabidopsis (*Arabidopsis thaliana*) (Pons et al. 2001; Boonman et al. 2007). Thicker leaves often have more developed palisade and spongy mesophyll layers, which improve internal light transmission and support rapid photosynthetic rates (Kalve et al. 2014; Kong et al. 2016).

At the reproductive stage, chickpea preferentially increased leaf thickness in the upper canopy rather than producing additional vegetative‐type leaves, a strategy that reduces shading of lower canopy layers and increases canopy photosynthesis (Feng et al. 2022). Overall, increased leaf thickness may enhance canopy light‐use efficiency, carbon assimilation and ultimately crop yield. However, these responses should be interpreted as the combined outcome of developmental progression and increasing irradiance during plant growth under greenhouse conditions. Future work should aim to disentangle the relative contributions of environmental variation, particularly irradiance, and developmental progression.

### 4.2 No Trade‐Off Between PPUE and PRE

We detected no significant correlation between leaf PPUE and PRE (Figure 7), refuting our hypothesis of a trade‐off. Similarly, members of the Proteaceae in severely P‐impoverished habitats exhibit both high PPUE and high PRE (Denton et al. 2007; Guilherme Pereira et al. 2019; Hayes et al. 2018), and recent work in Australian woody species also shows a positive correlation between these traits (Tsujii et al. 2024). Collectively, these observations suggest that leaf PPUE and P conservation can operate synergistically rather than in an antagonistic manner. The absence of a trade‐off may arise from temporal and compositional differences underlying these traits. PPUE affects short‐term physiological status at full leaf expansion and is influenced by P allocation among cellular pools (Guilherme Pereira et al. 2019; Hayes et al. 2018), particularly nucleic acid and metabolite P (Chen et al. 2026; Shen et al. 2024; Tsujii et al. 2024). In contrast, PRE integrates P dynamics over the lifespan of the leaf, during which senescence duration affects P remobilisation. Importantly, nucleic acid P—a key determinant of PPUE (Chen et al. 2026; Liang et al. 2026)—is largely resorbed during senescence (Suriyagoda et al. 2022; Tsujii et al. 2017), consistent with the high PRE we observed in this study (Figure 4; Figure S3). Thus, variation in PPUE need not constrain PRE, allowing these traits to vary independently.

### 4.3 Enhanced PRE Is Associated With Acid Phosphatase Activity, Sink Demand and Transpiration

Our second hypothesis was partially supported: leaf PRE increased during the reproductive stage and was coordinated with enhanced root P acquisition, while PPUE was stable. Three mechanisms likely contributed to the observed increase in PRE. First, elevated leaf acid phosphatase activity was associated with greater PRE and possibly with enhanced hydrolysis of organic P during leaf senescence (Figure 3). Second, the development of reproductive sinks increased P demand, which may have contributed to enhanced P reallocation. Third, leaf transpiration was positively associated with PRE (Figure 7; Table 2). Although the underlying mechanism remains uncertain, hydraulic interactions between xylem and phloem may facilitate phloem‐mediated P transport to reproductive organs (Hölttä et al. 2006; Pantin et al. 2012).

Interestingly, increased leaf thickness and LMA are usually associated with prolonged leaf lifespan (Wright et al. 2004; Yu et al. 2022); yet, in our study, thicker leaves coincided with reduced longevity. Despite the shorter senescence window, high PRE was achieved, potentially reflecting elevated acid phosphatase activity and other processes involved in P remobilisation during senescence (Veneklaas 2022; Figure 5). Investigating the gene regulatory networks and hormonal dynamics governing leaf senescence will be critical to understanding how efficient P remobilisation occurs within shortened timeframes.

### 4.4 Developmental Increases in Root P Acquisition and Leaf P Resorption

Chickpea simultaneously enhanced both external P acquisition and internal P conservation across developmental stages. Our previous study (Feng et al. 2026) showed that chickpea accessions exhibiting greater carboxylates recovered from the rhizosheath at the seedling stage also tend to exhibit greater PRE, suggesting a potential association between these traits at the genotypic level. However, that study focused on genotypic variation, whereas the present study demonstrates coordinated changes in both carboxylates recovered from the rhizosheath and PRE across developmental stages. These findings contrast with earlier studies proposing a trade‐off (Deng et al. 2016; Kou et al. 2017; Raven et al. 2018; Wright and Westoby 2003) or a “profit‐maximising” strategy favouring a single nutrient‐acquisition pathway (Bloom et al. 1985; Reichert et al. 2022). Some studies, however, support our results, showing simultaneous expression of both strategies in Australian woody shrubs (de Campos et al. 2013), boreal peatland communities (Ji et al. 2025), and *Quercus acutissima* populations along China's North–South Transect (Tong et al. 2022). Several mechanisms may account for this observed coordination between P‐acquisition and P‐conservation strategies. First, phosphatase synthesis requires N, and nitrogen limitation may increase the relative cost of enzyme production, potentially constraining P resorption from senescing leaves. Under such conditions, plants may rely more heavily on root P acquisition to meet their P demand (Estiarte et al. 2023; Raven et al. 2018; Wright and Westoby 2003). However, N_2_‐fixing plants, such as chickpea, may partially alleviate this constraint through biological nitrogen fixation (Feng et al. 2026). Second, flexible resource allocation among organs and developmental stages may further enhance nutrient‐use efficiency. For example, plants can alleviate nutrient limitation in arid–hot grassland by allocating less biomass to stems and relatively more to leaves (Yan et al. 2016), suggesting that flexible biomass partitioning can support nutrient‐use efficiency through adjusted organ‐level investment. Third, our findings suggest a significant role of leaf transpiration in mediating this observed coordination. As noted above, increased leaf transpiration may enhance phloem loading and nutrient remobilisation from senescing vegetative tissues to developing organs, thereby promoting internal P conservation. Concurrently, faster transpiration rates may enhance P transport in the soil–plant continuum by increasing mass flow of soluble P towards the root surface, whereas carboxylates recovered from the rhizosheath increase the mobilisation of sparingly‐soluble soil P into the soil solution (Cramer et al. 2008; Pang et al. 2018). Together, these complementary processes may enhance overall P availability and uptake by roots. Future research integrating the characterisation of P transporters and metabolic regulators in both roots and leaves may provide deeper insights into the genetic basis of these coordinated responses and support the development of P‐efficient crop cultivars. In addition, our findings suggest that transpiration—an important yet historically underestimated factor—should be considered not only in root P acquisition (Alam et al. 2026; Zou 2026), but also in leaf P resorption.

## 5 Conclusions

Our study demonstrates that chickpea exhibits pronounced plasticity in P‐use strategies across developmental stages. During reproduction, plants significantly increased leaf thickness and leaf mass per area, accompanied by enhanced leaf area‐based photosynthetic capacity, collectively improving canopy carbon assimilation. We found no evidence for a trade‐off between leaf PPUE and PRE. Instead, PRE increased substantially during reproduction while PPUE was stable, indicating that P‐use and P‐conservation strategies can operate independently. Enhanced PRE was associated with elevated leaf acid phosphatase activity, increased reproductive sink demand, and accelerated senescence, demonstrating that efficient internal P remobilisation can occur within a shortened timeframe. Reproductive development was also accompanied by increased root P acquisition, as reflected by greater carboxylates recovered from the rhizosheath and rhizosheath phosphatase activity. These coordinated above‐ and belowground responses indicate that chickpea can concurrently enhance external P acquisition and internal P recycling rather than prioritising a single strategy. Elevated leaf transpiration was associated with both carboxylates recovered from the rhizosheath and leaf P resorption, indicating a potential physiological association between belowground P acquisition and aboveground P remobilisation. Collectively, this coordinated enhancement of root P acquisition and leaf P resorption represents an efficient P‐use strategy during reproduction. These findings provide a physiological framework for improving plant P‐use efficiency by simultaneously enhancing belowground P acquisition and aboveground internal P recycling, offering potential guidance for breeding P‐efficient chickpea cultivars.

## Conflicts of Interest

The authors declare no conflicts of interest.

## Supporting information


Supporting File

## Data Availability

Data are publicly available at https://github.com/Franke-ovo/Vegetative_reproductive.
