# Supplementary material for: Concurrent Increases in Root Phosphorus Acquisition and Leaf Phosphorus Resorption Underpin Enhanced Phosphorus Economy in Chickpea During Reproductive Development
Source: Plant Cell Environ. 2026 Aug 9;49(11):9085–100. doi: 10.1111/pce.70797 (PMC13646661; doi:10.1111/pce.70797)
Supplement: Supplementary file 1 [file PCE-49-9085-s001.docx]

**Concurrent increases in root phosphorus acquisition and leaf phosphorus resorption underpin enhanced phosphorus economy in chickpea during reproductive development**

Xiaolong Feng^1,2*^, Shuyan Li^1,2^, Jiayin Pang^1,2*^, Peta L. Clode^1,3^, Hans Lambers^1,2^, Kadambot H. M. Siddique^2^

*Correspondence:

[xiaolong.feng@research.uwa.edu.au](mailto:xiaolong.feng@research.uwa.edu.au)

jiayin.pang@uwa.edu.au

**Supplementary captions :**

**Table S1.** Detailed information on five chickpea accessions used in the experiment.

**Table S2.** Reproductive traits and plant height of five chickpea accessions.

**Table S3.** Model comparison statistics testing the effect of developmental stage on leaf-trait variation using linear mixed-effects models.

**Figure S1.** Representative images of chickpea intact root systems: scanned root at the vegetative stage (left, six weeks after sowing) and photographed at the reproductive stage (right, 12 weeks after sowing).

**Figure S2.** Root nutrient concentrations of five chickpea accessions at the vegetative (blue, six weeks after sowing) and reproductive stage (red, 12 weeks after sowing) in five chickpea accessions under moderate P deficiency (25 mg P kg^–1^).

**Figure S3.** Phosphorus (P)-resorption efficiency of five leaf P fractions at the vegetative (six weeks after sowing) and reproductive (12 weeks after sowing) stage.

**Table S1.** Detailed information on five chickpea landraces used in the experiment.

| Accession | Origin | Market type | PRE |
| --- | --- | --- | --- |
| ICC 13219 | Iran | Desi | High |
| ICC 7184 | Turkey | Desi | High |
| ICC 9590 | Egypt | Desi | Low |
| ICC 7323 | Russia | Pea-shaped | Low |
| ICC 10885 | Ethiopia | Kabuli | Low |

PRE, leaf phosphorus-resorption efficiency.

**Table S2.** Reproductive traits and plant height of five chickpea accessions.

Data are means ± SE (n=5).

| Traits | ICC 13219 | ICC 7184 | ICC 9590 | ICC 7323 | ICC 10885 |
| --- | --- | --- | --- | --- | --- |
| DW_seed_ (g) | 22.2 (0.6)^ab^ | 23.2 (0.3)^a^ | 20.4 (0.6)^b^ | 23.0 (0.6)^a^ | 22.8 (0.5)^a^ |
| P_seed_ (mg g^-1^) | 2.13 (0.11)^a^ | 1.72 (0.05)^b^ | 2.06 (0.04)^a^ | 1.66 (0.08)^b^ | 1.65 (0.05)^b^ |
| TP_seed_ (mg) | 47.1 (1.4)^a^ | 40.0 (1.1)^b^ | 41.9 (1.7)^ab^ | 38.1 (1.7)^b^ | 37.7 (0.8)^b^ |
| DF (DAS) | 53.2 (0.7)^c^ | 71.6 (0.5)^a^ | 46.6 (0.5)^d^ | 60.0 (0.5)^b^ | 53.2 (0.5)^c^ |
| Podding (DAS) | 60.2 (0.6)^d^ | 76.6 (0.5)^a^ | 64.4 (0.8)^c^ | 67.6 (0.2)^b^ | 66.6 (0.5)^bc^ |
| Plant height (cm) | 51.8 (0.6)^e^ | 74.4 (0.5)^c^ | 82.0 (1)^b^ | 92.2 (1.4)^a^ | 57.4 (1.4)^d^ |

Abbreviations: DW_seed_, dry weight of seed, P_seed_, seed phosphorus concentration, TP_seed_, total seed phosphorus content; DF, flowering date (days after sowing, DAS); podding, podding date (DAS). Different letters indicate significant difference among accessions for each parameter (*p* < 0.05, Tukey’s test)

**Table S3.** Model comparison statistics testing the effect of developmental stage on leaf-trait variation using linear mixed-effects models. The null model = Trait ~ 1 +(1|Accession), the tested model = Trait ~ Stage +(1|Accession). Significant P values (*p* < 0.05) are highlighted in bold.

| Trait | AIC_null | AIC_stage | logLik_null | logLik_stage | Chisq | *P* value |
| --- | --- | --- | --- | --- | --- | --- |
| Chlorophyll | 5.12 | 1.68 | 0.44 | 3.16 | 5.45 | **0.019592** |
| Carotenoids | –118.86 | –148.65 | 62.43 | 78.33 | 31.79 | **1.71E-08** |
| LMA | 251.52 | 189.61 | –122.76 | –90.81 | 63.91 | **1.3E-15** |
| LDMC | –216.94 | –236.26 | 111.47 | 122.13 | 21.33 | **3.87E-06** |
| Leaf thickness | 363.61 | 313.95 | –178.81 | –152.97 | 51.66 | **6.59E-13** |
| E | 168.73 | 122.81 | –81.37 | –57.40 | 47.92 | **4.43E-12** |
| A_area_ | 252.36 | 230.91 | –123.18 | –111.45 | 23.45 | **1.28E-06** |
| A_mass_ | –41.90 | –39.92 | 23.95 | 23.96 | 0.02 | 0.886002 |
| g_sw_ | –34.41 | –50.84 | 20.20 | 29.42 | 18.43 | **1.76E-05** |
| WUE_i_ | 374.78 | 371.71 | –184.39 | –181.86 | 5.07 | **0.024397** |
| P_g_ | 18.63 | 11.04 | **–**6.32 | –1.52 | 9.60 | **0.001948** |
| P_s_ | 5.66 | –23.50 | 0.17 | 15.75 | 31.16 | **2.38E-08** |
| PRE | 317.45 | 286.18 | –155.72 | –139.09 | 33.26 | **8.05E-09** |
| APase | –42.27 | –69.68 | 24.14 | 38.84 | 29.41 | **5.86E-08** |
| N_g_ | 242.06 | 228.16 | –118.03 | –110.08 | 15.90 | **6.68E-05** |
| N_s_ | 174.38 | 175.52 | –84.19 | –83.76 | 0.85 | 0.355488 |
| NRE | 254.99 | 255.20 | –124.50 | –123.60 | 1.80 | 0.179917 |
| PPUE | 449.96 | 450.38 | –221.98 | –221.19 | 1.58 | 0.208694 |
| PNUE | 207.62 | 205.99 | –100.81 | –99.00 | 3.63 | 0.056838 |
| Zn | 381.20 | 308.80 | –187.60 | –150.40 | 74.40 | **6.39E-18** |

Abbreviations: APase, leaf acid phosphatase activity; LMA, leaf mass per area; LDMC, leaf dry matter content; NRE/PRE, nitrogen/phosphorus resorption efficiency; PNUE/PPUE photosynthetic nitrogen/phosphorus-use efficiency; E, transpiration rate; g_sw,_ stomatal conductance; A_area_/A_mass_ photosynthetic rate based on area or mass; WUE_i_, intrinsic water-use efficiency; chlorophyll/carotenoids, chlorophyll or carotenoids concentration; Zn, leaf zinc concentration. *p* < 0.05 was bold.

AIC null, Akaike Information Criterion for the null model, AIC_stage, AIC for the model including growth stage, logLik_null, Log-likelihood of the null model, logLik_stage, Log-likelihood of the model with growth stage, Chisq, likelihood ratio chi-square statistic.

**Figure S1.** Representative images of chickpea intact root systems: scanned root at the vegetative stage (left, six weeks after sowing) and photograph at the reproductive stage (right, 12 weeks after sowing).

**Figure S2.** Root (a) phosphorus (P) and (b) nitrogen (N) concentration of five chickpea accessions at the vegetative (blue, six weeks after sowing) and reproductive stage (red, 12 weeks after sowing) in five chickpea accessions under moderate P deficiency (25 mg P kg^–1^). Data are means ± SE (n = 4). Tukey’s test detected differences among accessions at each growth stage. Asterisks denote differences between growth stages for the same accession: * *p* < 0.05, ** *p* < 0.01, *** *p* < 0.001 (*t*-test). Non-significant differences are not shown.

**Figure S3.** Phosphorus-resorption efficiency of five leaf phosphorus (P) fractions at the vegetative (six weeks after sowing) and reproductive stage (12 weeks after sowing ). Error bars represent 95% confidence intervals from 10,000 bootstrap resamples. Data are the same as in Figure 4, shown here for clarity.
